# Maksymilian Rose (1883–1937)

**DOI:** 10.1007/s00415-017-8592-y

**Published:** 2017-08-18

**Authors:** Katarzyna Pekacka-Falkowska, Michal K. Owecki, Anna Maria Pekacka

**Affiliations:** 1 Department of History of Medical Sciences, Poznan University of Medical Sciences, Przybyszewskiego 37a, 60365 Poznan, Poland; 2 Department of Neurorehabilitation, Zürcher Reha Zentrum Wald, Faltigbergstrasse 7, 8636 Wald, Switzerland

The year 2017 marks the 80th anniversary of the death of Maksymilian Rose, one of the pioneers of brain cytoarchitectonics.

Rose was born on May 19, 1883 into a Jewish low-income family in Przemyśl, Galicia, a Polish territory annexed by the Habsburg Empire (Austro-Hungary). After completing his secondary education in the small town of Wadowice, he studied medicine at the Jagiellonian University (JU), graduating with distinction in 1908. Next he took up a position at Cracow’s University Neurology and Psychiatry Department where he worked as an assistant of Professor Jan Pilz until 1910. The young professional quickly gained his new superior’s recognition, which enabled him to be trained in the best neurological centres of the time in Western Europe. Therefore, Rose spent the following two years abroad, continuing his education at various renowned clinics in Switzerland and Germany. In Berlin, he studied under Herman Oppenheim and Theodor Ziehen, the leading neurologists and psychiatrists in the German Empire. Shortly thereafter he moved to the Rheinau psychiatric clinic and, in 1912, to Professor Robert Gaupp’s clinic in Tübingen, where he worked on cytoarchitectonics under Professor Korbinian Brodmann who played a decisive role in shaping his further research interests [1].

After returning to Cracow in 1913, Rose was accepted for a position at the JU Descriptive Anatomy Unit where he began his own research on the cytology of neurons in the cerebral cortex. Some of his works from that period of time covered such topics as cytoarchitectonic parcellation of forebrain of reptiles and birds [2]. Yet, it was *On the histological principle of divisions of the cerebral cortex* that earned him international attention [3]. Consequently, in 1925, he was appointed by Professor Oskar Vogt, the prospective director of the neurology department at Kaiser Wilhelm Institute in Berlin for Anthropology, Human Heredity and Eugenics. Rose remained there three busy years as a Member of the Institute’s Scientific Board. In 1926, he served on the editorial board of “Journal für Psychologie und Neurologie”, a leading neurological journal in Germany at that time, and in 1927, on Vogt’s request, he was appointed as its editor-in-chief. At the same time, he worked on two atlases on cerebral cortex cytology and cytoarchitectonics [4, 5]. He also proved the division of the retrosplenial cortex into the granular and agranular part [6] and provided a detailed cytoarchitectonic analysis of the insula [7].

In 1928, Rose moved to the University of Warsaw, despite strong protests of his German colleagues, Walther Spielmeyer and Oskar Vogt. There he completed his habilitation thesis and two years later, in 1930, he founded the first Polish Brain Research Institute. In the newly established centre Rose introduced a modern neuropathological staining technique—the Cajal’s method.

In 1931, Rose was appointed as the chair at the Psychiatry Department of the University of Vilnius to succeed Stanisław Władyczko, a prominent figure in central European psychiatry. In interwar Poland, only very few Poles of Jewish origin succeeded in attaining the position of a full professor; thus Rose decided to leave Warsaw, transferring the Brain Research Institute with him. In Vilnius he continued his broad scientific research, successfully joining it with his teaching duties and directing the neurological clinic. He also maintained very close contacts with the scientific community in German-speaking lands, first and foremost with Professor Vogt, who trained Rose and finally became his friend.

Rose published dozens of scientific works in Polish, German and Spanish, in the majority on the anatomy and cytoarchitecture of the brain. He focused his scientific attention on the neurology and neuroanatomy borderline, devoting himself to research on embryology of the nervous system as well as cytological and morphological structure of the brain. Worth noting, Rose and his close collaborator Max Bielschowsky invented an innovative and brilliant method of selective staining of the V and VI layers of the cerebral cortex with use of indophenol [8]. Rose reported the method in 1927 as a member of the scientific staff of Kaiser Wilhelm Institute for Brain Research. In his publications, Rose discussed the major questions of neuroanatomy and phylogenesis of the nervous system.

The importance of Rose for the scientific community was proved as he was invited to prepare a part of a neurology manual edited by Oswald Bumke and Otfrid Foerster, both of whom hold prominent positions in the history of European neurology [9]. In the book printed in 1935, he broadly presented the issues of cytostructure and ontogenesis of the central nervous system. He concentrated not only on the brain comparative anatomy, but also analysed characteristic changes of the brain cortex cytostructure, developing in a number of neurological disorders. Thus, Rose was a recognized pioneer researcher of biochemical activity of the brain cortex cells, both in physiological and pathological states. Based on his own works he established an original classification of the brain cortex areas, dependent on morphological and phylogenetic criteria.

The crowning achievement of Rose’s scientific work was the cytoarchitectural examination of the brain of Marshal Pilsudski, a political leader of the Second Polish Republic [10]. Rose took pride in this work, but he did not succeed in completing it. He died suddenly in 1937 from a heart attack, during a short break between his lectures on psychiatry, at the peak of his scientific career. In a funeral eulogy, Wóycicki, the Rector of the University of Vilnius stated that “the death of Maximilian Rose is a profound loss for a scientific community; we lost a man of a superior intellect and with a good heart”.

## References

[1] Rose M (1912). Histologische Lokalisation der Großhirnrinde bei kleinen Säugetieren (Rodentia, Insectivora, Chiroptera). Journal für Psychologie und Neurologie.

[2] Rose M (1914). Über die cytoarchitektonische Gliederung des Vorderhirns der Vögel. Journal für Psychologie und Neurologie.

[3] Rose M (1925/1926) Über das histogenetische Prinzip der Einteilung der Großhirnrinde. Journal für Psychologie und Neurologie 32: 97–160

[4] Rose M (1929). Cytoarchitektonischer Atlas der Großhirnrinde der Maus. Journal für Psychologie und Neurologie.

[5] Rose M (1931). Cytoarchitektonischer Atlas der Großhirnrinde des Kaninchens. Journal für Psychologie und Neurologie.

[6] Rose M (1927). Gyrus limbicus anterior und Regio retrosplenialis (Cortex holoprotoptychos quinquestratificatus) vergleichende Architektonik bei Tier und Mensch. Journal für Psychologie und Neurologie.

[7] Rose M (1928). Die Inselrinde des Menschen und der Tiere. Journal für Psychologie und Neurologie.

[8] Bielschowsky M, Rose M (1927). Die Bedeutung des Nachweises oxydierender und reduzierender Gewebefermente für Lokalisationsfragen des Gehirns. Journal für Psychologie und Neurologie.

[9] Rose M (1935) Cytoarchitektonik und Myeloarchitektonik der Großhirnrinde. In: O. Bumke, O. Foerster (Hrsg.) Handbuch der Neurologie, Allgemeine Neurologie. 1. Anatomie. Springer, Berlin, pp 588–778

[10] Rose M (1938) Le cerveau de Joseph Pilsudski. Première Partie. J. Zawadzki: Wilno

